# Assessment of *Streptococcus pneumonia*e pilus islet-1 prevalence in carried and transmitted isolates from mother–infant pairs on the Thailand–Burma border

**DOI:** 10.1111/j.1469-0691.2011.03711.x

**Published:** 2011-10-31

**Authors:** P Turner, S Melchiorre, M Moschioni, M A Barocchi, C Turner, W Watthanaworawit, N Kaewcharernnet, F Nosten, D Goldblatt

**Affiliations:** 1 Shoklo Malaria Research UnitMae Sot; 2 Mahidol-Oxford Tropical Medicine Research UnitBangkok, Thailand; 3 Centre for Tropical Medicine, University of OxfordOxford, UK; 4 Novartis Vaccines and Diagnostics s.r.l.Siena, Italy; 5 Immunobiology Unit, Institute of Child Health, University of LondonLondon, UK

**Keywords:** Carriage, carriage duration, colonization, PI-1, pilus-1, *Streptococcus pneumoniae*, transmission

## Abstract

*Streptococcus pneumoniae* pilus islet-1 (PI-1)-encoded pilus enhances *in vitro* adhesion to the respiratory epithelium and may contribute to pneumococcal nasopharyngeal colonization and transmission. The pilus subunits are regarded as potential protein vaccine candidates. In this study, we sought to determine PI-1 prevalence in carried pneumococcal isolates and explore its relationship with transmissibility or carriage duration. We studied 896 pneumococcal isolates collected during a longitudinal carriage study that included monthly nasopharyngeal swabbing of 234 infants and their mothers between the ages of 1 and 24 months. These were cultured according to the WHO pneumococcal carriage detection protocol. PI-1 PCR and genotyping by multilocus sequence typing were performed on isolates chosen according to specific carriage and transmission definitions. Overall, 35.2% of the isolates were PI-1-positive, but PI-1 presence was restricted to ten of the 34 serotypes studied and was most frequently associated with serotypes 19F and 23F; 47.5% of transmitted and 43.3% of non-transmitted isolates were PI-1-positive (OR 1.2; 95% CI 0.8–1.7; p 0.4). The duration of first-ever infant pneumococcal carriage was significantly longer with PI-1-positive organisms, but this difference was not significant at the individual serotype level. In conclusion, PI-1 is commonly found in pneumococcal carriage isolates, but does not appear to be associated with pneumococcal transmissibility or carriage duration.

## Introduction

*Streptococcus pneumoniae* is a significant global pathogen [1]. Additionally, pneumococcus is a nasopharyngeal commensal, and infants are frequently colonized [2,3]. Various cell surface components, including pneumococcal surface adhesin A, choline-binding protein A, pneumococcal serine-rich repeat protein, and pneumococcal adherence-virulence factor A, have been shown to contribute to nasopharyngeal adherence and colonization, but their mechanisms of action remain incompletely understood [3–7]. The RrgA subunit of the surface exposed pilus-1 filamentous structure also enhances pneumococcal adherence to respiratory epithelial cells *in vitro* [8,9]. Pneumococcal pilus-1 is encoded by the pilus islet-1 (PI-1; *rlrA* islet), and is composed of three subunits: RrgA, RrgB, and RrgC [9–11]. As immunization with pilus antigens is protective against lethal intraperitoneal challenge in a mouse model, pilus subunits are regarded as potential candidates for inclusion in a protein-based pneumococcal vaccine [12]. However, PI-1 presence is not universal: studies of predominantly invasive pneumococci have found that PI-1 is present in isolates from a limited number of serotypes (particularly those included in the pneumococcal seven-valent conjugate vaccine (PCV7)) and that its presence correlates with genotype by multilocus sequence typing (MLST) [13–18]. Given the prevalence of asymptomatic colonization, and the fact that the serotype/genotype structure of invasive pneumococci is not stable [19], further work to describe the prevalence and function of pilus-1 in pneumococcal carriage strains is warranted.

In this study, our aims were to: (i) determine PI-1 prevalence in pneumococcal carriage isolates from infants and their mothers; (ii) determine which PI-1-positive clones were associated with carriage in the study region; and (iii) explore the possible functions of the pilus *in vivo*, and in particular evaluate whether PI-1 presence had an effect on pneumococcal transmission or carriage duration.

## Materials and Methods

### Pneumococcal isolates

The isolates characterized in this work were collected from 2007 to 2010, during a longitudinal carriage and pneumonia study of 965 infants at Maela refugee camp, Thailand. Maela was established in 1984, and *c.* 40 000 Burmese refugees, predominantly of Karen ethnicity, live in a 4-km^2^ area. Pneumococcal vaccines are not available in the camp. Nested within the study was a subcohort of 234 mother–infant pairs who were studied in more detail: between 1 and 24 months of age, these infants had a nasopharyngeal swab (NPS) and blood taken at monthly surveillance visits, and their mothers had an NPS taken at the same time. NPSs were processed according to the WHO pneumococcal colonization protocol, as previously described [20,21]. All morphologically distinct pneumococcal isolates from each swab culture were serotyped by latex agglutination [22]. Non-typeable (NT) isolates, either morphologically typical pneumococcal colonies (probably encapsulated organisms) or rough colonies (probably non-encapsulated organisms) that were non-reactive with typing antisera, were confirmed by bile solubility and absent capsular swelling with Omniserum (Statens Serum Institute, Hillerod, Denmark). Serotype 6C was identified by PCR [23].

Mother and infant pneumococcal carriage patterns for the first 12 months of follow-up were reviewed, and isolates were chosen for further analysis according to the definitions described below.

### Definition of transmission and carriage

Transmission of a pneumococcus was defined as the presence of an identical pneumococcal serotype and MLST genotype in both mother and infant at the same visit (‘concordant transmission’) or when the pneumococcus was isolated from the mother and the infant during a ‘transmissibility episode’ but not at the same visit (‘discordant transmission’). A non-transmitted pneumococcus was a carried serotype that never appeared in the other member of the mother–infant pair during the transmissibility episode as defined below (Fig. S1). Transmissibility episodes were identified by carriage within a mother–infant pair when there was the same pneumococcal serotype cultured from two or more NPSs (from mother and/or infant), separated by two or fewer NPSs negative for that serotype. For serotypes 1 and 5, which are known to be carried for very short durations, a single positive NPS could define non-transmission.

For the determination of carriage duration in infants, a pneumococcal acquisition was defined as midpoint between the last negative swab and the first positive swab for a given serotype. Termination of the carriage episode was similarly defined as the midpoint between the first of two consecutive negative swabs and the last positive swab for the serotype.

### PI-1 detection

PI-1 presence was determined as previously described [14]. Briefly, PCRs were performed directly from bacteria, with the primers listed in Table S1. Primers were designed on conserved regions on the boundaries of PI-1 (459for and 470rev) and within PI-1 (P01rev, P11for, P08for, and P08rev).

### MLST

MLST was performed as previously described [24]. Briefly, PCR amplifications were performed directly from the bacteria with the standard primer pairs. Sequences were obtained on both strands with an ABI 3730xl DNA Analyzer (Life Technologies Corporation, Carlsbad, CA, USA). Sequence type (ST) was determined by use of the MLST website (http://spneumoniae.mlst.net). eBURST (http://spneumoniae.mlst.net/eburst/) was run with default settings on the entire MLST database, and each ST was assigned to a clonal complex (CC) [25]. CCs were named in accordance with the ST number of the founder predicted by eBURST.

### Statistical analysis

Statistical analyses were carried out with STATA 10.1 (StataCorp, College Station, TX, USA). The chi-squared test, Fisher’s exact test and ORs were used to compare proportions. Carriage duration was estimated by survival analysis methods, with the log-rank test being used to compare groups [2].

### Study ethics

Ethical approval for the carriage study was granted by Mahidol University, Thailand and Oxford University, UK.

## Results

### Pneumococcal isolates and PI-1 prevalence

In the first 12 months of follow-up, 4921 surveillance NPS were collected from the 234 mother–infant pairs (84.7% of expected). In total, 2497 isolates were cultured, and 896 (35.9%) of these were included in the current work. All isolates meeting the criteria described below were included, resulting in 34 serotypes being represented in the isolate selection. These serotypes accounted for 90.1% (2251/2497) of isolates in the entire cohort isolate collection.

**FIG. 1:**
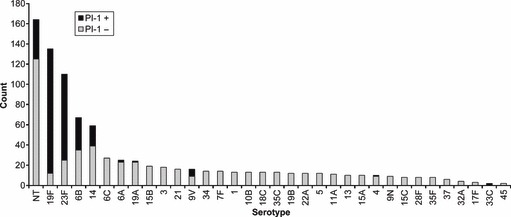
Pilus islet-1 (PI-1) presence by serotype in 887 carried pneumococcal isolates (includes all commonly carried serotypes).

We successfully determined the PI-1 status of 887/896 (98.9%) isolates: 35.2% (312/887) were positive. PI-1-positive isolates were restricted to ten serotypes: 4, 6A, 6B, 9, 14, 19F, 19A, 23F, 33C, and NT (Fig. 1).

### PI-1 and transmission

To determine whether PI-1 presence had an effect on pneumococcal transmission within the mother–infant pairs, a panel of isolates was selected and analysed by both PI-1 PCR and MLST (to define genotype). We elected to study serotypes contained in, or related to, current conjugate vaccines (PCV13 + 6C), as well as NT isolates, which were overrepresented in our population (the commonest pneumococcal ‘type’ identified from mother swabs, and the third commonest in infants). In the first year of follow-up, these serotypes accounted for 69.6% and 59.8% of isolates carried by infants and mothers, respectively. In the case of a potentially transmitted pneumococcal serotype, i.e. isolates carried by both mother and infant at the same visit, or at sequential visits, single isolates from both mother and infant were selected for study. For non-transmitted isolates, i.e. isolates carried by only the mother or infant, a single isolate was studied. Additionally, we analysed every isolate from carriage episodes of all serotypes in eight mother–infant pairs to determine the clonality of pneumococcal carriage.

**FIG. 2:**
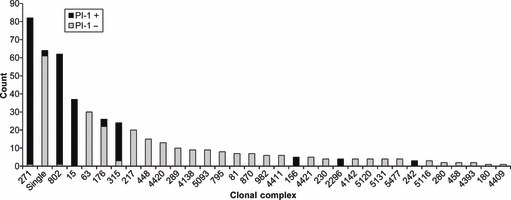
Pilus islet-1 (PI-1) presence by clonal complex in 483 carried pneumococcal isolates (pneumococcal 13-valent conjugate vaccine serotypes plus 6C and non-typeable (NT) only).

Of the 489 isolates selected for the primary analysis, PI-1 PCR was uninterpretable in six, resulting in 483 analysable isolates. Overall, 219/483 (45.3%) of these were PI-1 positive. PI-1 was found in NT isolates and in 6/14 serotypes studied, all of which, apart from one 19A isolate, were PCV7 serotypes (Table 1). PI-1 was found most frequently in 19F (91%) and 23F (77%) isolates, although these serotypes, according to the MLST and eBURST analyses, were dominated by a single CC each (19F, CC271; 23F, CC802). Overall, PI-1-positive isolates were members of a restricted group of CCs (Fig. 2).

**TABLE 1:** Pneumococcal serotype distribution and isolate transmission category in mother–infant pairs (pneumococcal 13-valent conjugate vaccine serotypes plus 6C and non-typeable (NT))

Serotype	Total, *N*	Transmitted^a^, *N* (% within serotype)	PI-1 present, *N* (% within serotype)
1	12	4 (33.3)	0 (0.00)
3	11	4 (36.4)	0 (0.00)
4	4	2 (50.0)	0 (0.00)
5	10	2 (20.0)	0 (0.00)
6A	16	6 (37.5)	0 (0.00)
6B	54	27 (50.0)	29 (53.7)
6C	15	4 (26.7)	0 (0.00)
7F	5	2 (40.0)	0 (0.00)
9V	8	4 (50.0)	5 (62.5)
14	46	28 (60.9)	15 (32.6)
18C	7	4 (57.1)	0 (0.00)
19F	89	50 (56.2)	81 (91.0)
19A	14	4 (28.6)	1 (7.1)
23F	82	38 (46.3)	63 (76.8)
NT	110	57 (51.8)	25 (22.7)
Total	483	236 (48.9)	219 (45.3)

PI-1, pilus islet-1.

a Concordant and discordant transmission combined.

With serotyping of a representative isolate alone, 253/483 (52.4%) isolates would have been classified as transmitted (153 concordant; 100 discordant) and 230/483 (47.6%) as non-transmitted. With a combination of MLST and serotype, nine concordant and eight discordant ‘transmitted’ isolates (17/483; 3.5%) were reclassified as non-transmitted, resulting in final totals of 236/483 (48.9%) transmitted and 247/483 (51.1%) non-transmitted isolates. PI-1 presence was not correlated with transmission: 47.5% (112/236) of transmitted and 43.3% (107/247) of non-transmitted isolates were PI-1 positive, with an OR of 1.2 (95% CI 0.8–1.7; p 0.4), and no significant differences were seen at the individual serotype level.

We analysed an additional 91 isolates, from seven mother–infant pairs and one mother–twin infant unit, to confirm that sequential isolates of the same serotype in an individual, and isolates of the same serotype in a mother–infant pair, were indeed identical. In all mother–infant pairs, each serotype carried was represented by a single ST per carriage/transmission episode, with three exceptions (Table S2).

### PI-1 and first pneumococcal carriage episode duration in infants

To analyse the correlation between PI-1 presence and the duration of infant carriage, we determined the PI-1 status of 316 isolates from the first carriage episodes of all common serotypes (defined as serotypes with at least ten carriage episodes). These episodes included first episodes of carriage of each serotype, not only an infant’s first ever pneumococcal carriage episode. We inferred the PI-1 status for the entire carriage episode from the PI-1 PCR result of a single isolate.

Given the well-described association of shorter carriage in individuals with prior pneumococcal exposure, we subsequently focused our analysis only on first-ever episodes of carriage (i.e. one per infant) [2]. From the 316 carriage episodes selected, we identified 216 analysable first-ever carriage episodes: PI-1 PCR data were available for at least one isolate in 180 of these (90 pneumococcal 13-valent conjugate vaccine serotypes, 58 non-vaccine serotypes, and 32 NT pneumococci). Median and mean durations of the first pneumococcal carriage episode were 63 days (95% CI 61–91) and 105 days (95% CI 91–119), respectively, but varied considerably by serotype (Table 2). On comparison of the 180 carriage episodes where PI-1 PCR results were available, PI-1-positive carriage episodes (51/180; 28.3%) were significantly longer than those associated with a PI-1-negative organism (median 152 days (95% CI 93–213) vs. 61 days (95% CI 57–90); mean 177 days (95% CI 137–218) vs. 84 days (95% CI 72–97); p <0.0001). However, the analysis of carriage duration was confounded by serotype. In our study, serotypes 19F and 23F had the longest duration of carriage, and were both predominantly PI-1-positive. To analyse the relative contributions of serotype and PI-1 to carriage duration, we fitted a Cox regression model and found that, on stratification of carriage by serotype, PI-1 presence was not associated with a significant change in carriage duration (hazard ratio 0.7 (95% CI 0.4–1.2; p 0.2)).

**TABLE 2:** First pneumococcal carriage episode duration (six commonest serotypes)

Serotype	Number of episodes	Median carriage duration (days) (95% CI)	Mean carriage duration (days) (95% CI)
6B	13	121 (90–153)	119 (93–145)
14	8	62 (30–151)	86 (45–128)
19F	21	213 (63–243)	231 (154–308)
23F	21	184 (62–277)	176 (124–229)
35F	9	121 (30–180)	124 (66–182)
NT	33	31 (31–61)	72 (44–99)

NT, non-typeable.

## Discussion

This study is the largest investigation of the prevalence and possible function of the PI-1-encoded pilus in carriage isolates of *S. pneumoniae*. It also provides pneumococcal strain data from Southeast Asia, a densely populated region that is under-represented in the pneumococcal carriage and disease epidemiology literature. As serotypes are commonly used as the basis for defining transmission, we were interested in the frequency with which a molecular analysis may confound the analysis of transmission. By genotyping, discrepancies were discovered in only 3.5% of transmission events classified by serotype. We also demonstrated that pneumococci of the same serotype are predominantly clonal within a discrete carriage episode. We therefore concluded that serotype can be used to define transmission events and carriage episodes with relatively high confidence, but that the inclusion of genotyping is vital to ensure complete accuracy.

Because of the selection criteria for the isolates included in the study, we cannot describe the overall pilus prevalence for the population. However, in the 887 carriage isolates analysed, the pilus prevalence was 35.2%. Among the 34 serotypes analysed, we found that PI-1 was present only in NT isolates and nine other serotypes: 65% of PCV7 isolates were PI-1-positive, as compared with 9% of non-vaccine serotypes. Interestingly, Basset *et al.*, examining nasopharyngeal and invasive pneumococcal isolates from the American Indian collection, also found that PCV7 strains were significantly more likely to be PI-1-positive than non-vaccine serotypes, but that the overall proportion of PI-1-positive isolates was slightly lower [13]. They, along with other authors, demonstrated that PI-1-positive strains were contained within a small number of CCs [14–16]. Indeed, in the current study, we demonstrated that PI-1-positive isolates were clustered predominantly within four dominant CCs (CC15, CC271, CC315, and CC802). The strong association between serotype/CC and pilus presence, along with strain selection criteria, and the different regional distribution of the clones, may explain the variability in PI-1 prevalence between different studies.

We did not demonstrate a pilus-attributable effect on pneumococcal transmissibility. However, there are limitations to our study that may be important confounders for this analysis. The carriage study was carried out in a densely populated refugee camp where 13% of the population are <5 years old, and there is likely to be frequent transmission of nasopharyngeal organisms both within families and between members of the community. Despite the use of a combination of serotype and ST to increase the accuracy of our transmissibility categories, we cannot exclude the possibility that ‘non-transmitted’ strains were effectively transmitted between mother or infant and others but not detected by us. Several studies have documented the clustering of pneumococcal serotypes and genotypes within families, which highlights the difficulty of assigning a definitive ‘non-transmitted’ label to isolates collected from an incomplete household group [26,27]. However, we focused on mother–infant transmission, because we felt that the absence of a strain in one member of this pair would be the best marker for relative non-transmissibility in the early months of life. In addition, we included both concordant and discordant time-point pneumococcal serotype/ST identifications in the mother–infant pair as ‘transmitted’, as long as they occurred no more than 2 months apart. However, the study sampling frequency may have been too low to demonstrate transmission of serotypes carried for very short durations. Also, in the presence of multiple serotype carriage, a common occurrence in infancy and one that is underestimated by standard culture protocols [21], a particular serotype may become undetectable for a period of time before re-emerging as the dominant serotype, and this may result in incorrect categorization regarding transmissibility.

Although, in the crude analysis, PI-1 presence was associated with longer first pneumococcal carriage episodes in infants, we could find no significant association between carriage duration and PI-1 at the individual serotype level. This is likely to be the result of the low numbers of carriage episodes of individual serotypes (resulting in wide CIs around the carriage duration estimates) and the restricted number of clones within each serotype. We could have increased the number of carriage episodes included in our analysis by looking at all carriage episodes of each serotype rather than restricting our investigation to each infant’s first-ever carriage episodes, but this would have introduced other confounding factors, such as the impact of previous carriage and immune factors on subsequent carriage episode duration. Therefore, it is possible that, as a result the study sample size, a small effect of pilus on either transmission or carriage duration may have been missed.

In conclusion, we found that Southeast Asian pneumococcal carriage isolates had a similar pilus prevalence to to that in previously described strain collections, which is helpful in the ongoing assessment of likely global coverage of a pilus subunit-containing vaccine. Despite its known role in pneumococcal attachment, we could not determine a clear impact of PI-1 presence on transmissibility or carriage duration.

## Supporting Information

**Figure S1.** Pneumococcal serotype transmission examples.

**Table S1.** PI-1 PCR primer set.

**Table S2.** Detailed study of pneumococcal carriage in the first year of life in eight mother-infant pairs.
