# Supplementary material for: Assessment of Streptococcus pneumoniae pilus islet-1 prevalence in carried and transmitted isolates from mother–infant pairs on the Thailand–Burma border
Source: Clin Microbiol Infect. 2011 Oct 31;18(10):970–5. doi: 10.1111/j.1469-0691.2011.03711.x (PMC3469734; doi:10.1111/j.1469-0691.2011.03711.x)
Supplement: Supplementary file 1 [file clm0018-0970-SD1.doc]

Figure S1. Pneumococcal serotype transmission examples***.*** (A) non-transmission of serotypes 19F and NT; (B) concordant transmission of serotype 6B; (C) discordant transmission of serotype 23F. Horizontal arrows represent an infant’s first year of life, with the numbers indicating age in months. Pneumococcal serotypes shown above these arrows represent carried isolates from the infant and those below the arrows are from the mother. Shading highlights the criteria used to select isolates for PI-1 PCR and MLST.


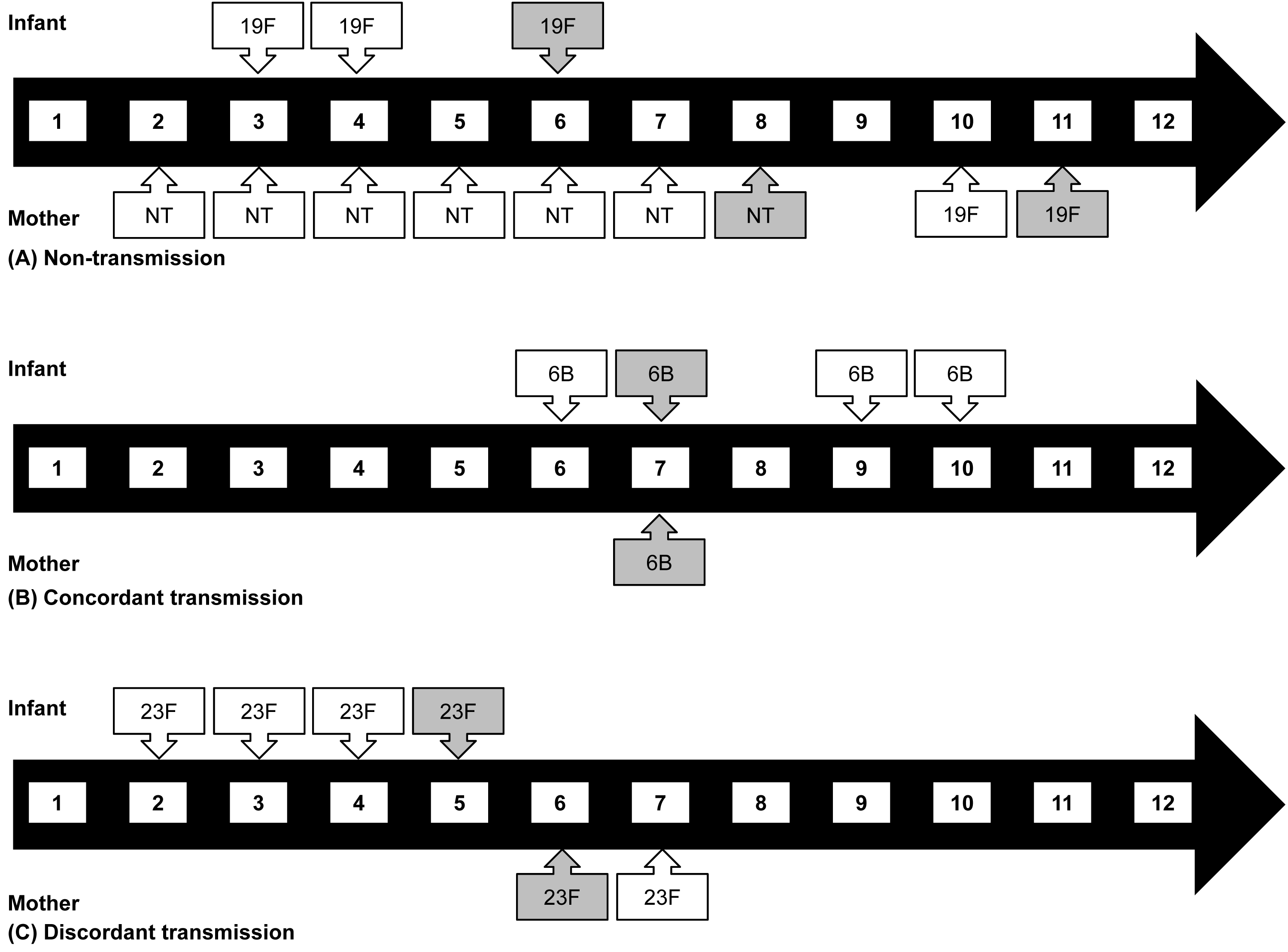


Table S1. PI-1 PCR primer set

| **Forward primer** | **Sequence (5’→3’)** | **Reverse primer** | **Sequence (5’→3’)** |
| --- | --- | --- | --- |
| 459 for | AACTGAATTGACACAACGTGTCTT | 470 rev | GCCACACAAGATGTTGATGCTTTT |
| 459 for | AACTGAATTGACACAACGTGTCTT | P01 rev | AGCGACAAGCCACTGTATCATATT |
| P08 for | TGAGATTTTCTCGTTTCTCTTAGC | P08 rev | AATAGACGATGGGTATTGATCATGT |
| P11 for | GCCATTTGGATCAGCTAAAAGTT | 470 rev | GCCACACAAGATGTTGATGCTTTT |

***Table S2. Detailed study of pneumococcal carriage in the first year of life in eight mother-infant pairs. Pneumococcal serotypes and their MLST genotype (in parentheses) are given for each nasopharyngeal sampling point.***

| **Infant age (m)** | | **1** | | **2** | | **3** | | **4** | | **5** | | **6** | | **7** | | **8** | | **9** | | **10** | **11** | **12** |
| --- | --- | --- | --- | --- | --- | --- | --- | --- | --- | --- | --- | --- | --- | --- | --- | --- | --- | --- | --- | --- | --- | --- |
| ***Pair 1*** | | | | | | | | | | | | | | | | | | | | | | |
| **Infant** |  | |  | | 19A  (230) | | 19A  (230) | | 14  (63) | | 14  (63) | | 14  (63) | | NT  (4133) | | 6C  (4420) | | 6C (4420)  NT (4133) | | 6C  (4420) | 6C  (4420) |
| **Mother** |  | |  | | 35F  (4418) | | 14  (63) | |  | |  | |  | |  | |  | |  | | 7F  (3545) | 7F  (3545) |
| ***Pair 2*** | | | | | | | | | | | | | | | | | | | | | | |
| **Infant** |  | | NT  (4136) | | NT  (4136) | | NT  (4136) | |  | | NT  (4136) | | NT  (4136) | | NT  (4136) | | NT  (4136) | | NT  (4136) | |  |  |
| **Mother** | NT  (4136) | |  | | 7F  (3545) | | 7F  (3545) | | NT  (4136) | | 7F  (3545) | | 7F  (3545) | | 7F (3545)  NT (4136) | | 7F  (3545) | | NT  (4136) | | NT  (4136) | NT  (4136) |
| ***Pair 3*** | | | | | | | | | | | | | | | | | | | | | | |
| **Infant** |  | | 14  (63) | | 14  (63) | | 14  (63) | | NT  (4451) | | 14  (63) | | 14  (63) | |  | | 19F  (81) | | 22A  (910) | | 19F  (81) | 22A  (910) |
| **Mother** | 32A (5092) | | 32A (5092) | | 32A (5092) | | NT  (5121) | | 14  (63) | | NT  (4451) | | 14  (4451) | | NT  (4451) | | 32A  (5092) | |  | | NT  (4451) | 19F  (81) |
| ***Pair 4*** | | | | | | | | | | | | | | | | | | | | | | |
| **Infant** |  | |  | | 19F  (4414) | | 19F  (4414) | | 19F  (4414) | | 19F  (4414) | | 19F  (4414) | | 19F  (4414) | | 23F  (802) | | 19F  (4414) | | 19F  (4414) | 23F  (802) |
| **Mother** | 37  (447) | | 37  (447) | |  | |  | | 37  (447) | | 37  (447) | |  | | 37  (447) | | 37  (447) | |  | |  | 19F  (4414) |
| **Infant age (m)** | | **1** | | **2** | | **3** | | **4** | | **5** | | **6** | | **7** | | **8** | | **9** | | **10** | **11** | **12** |
| ***Pair 5 (twins)*** | | | | | | | | | | | | | | | | | | | | | | |
| **Infant 1** | NT  (448) | | 33C  (5096) | | 33C  (5096) | | 17F  (5098) | | 21  (5103) | | 21  (5103) | | 19F  (5106) | | 19F  (5106) | | 19F  (4414) | | 19F  (4414) | | 6A  (4936) | 19F (4414)  NT (448) |
| **Infant 2** |  | | 17F  (5098) | | 21  (5103) | | 21  (5103) | | 17F  (5098) | | 21  (5103) | | 21  (5103) | | 21  (5103) | |  | | 19F  (4414) | | 6A  (4936) | 6A  (4936) |
| **Mother** | NT  (448) | |  | | 1  (217) | |  | |  | | NT  (448) | |  | |  | |  | |  | |  |  |
| ***Pair 6*** | | | | | | | | | | | | | | | | | | | | | | |
| **Infant** |  | | NT  (4133) | | NT  (4133) | | 19B  (5095) | | 1  (217) | | NT  (4133) | | 9V  (280) | | 9V  (280) | | 19F  (4414) | | 19F  (4414) | | 19F  (4414) | 19F  (4414) |
| **Mother** | 19B  (5095) | | 19B  (5095) | |  | |  | | NT  (4133) | |  | |  | | 9V  (5123) | | 9V  (280) | |  | |  |  |
| ***Pair 7*** | | | | | | | | | | | | | | | | | | | | | | |
| **Infant** | 45  (5097) | | 45  (5097) | | 23F  (802) | | 23F  (802) | | 23F  (802) | |  | | 6B  (315) | |  | | 23F  (802) | | 23F  (802) | | 23F  (802) |  |
| **Mother** |  | | 23F  (802) | |  | | 23F  (802) | | 23F  (802) | | 6B  (315) | |  | |  | |  | |  | | 23F  (802) |  |
| ***Pair 8*** | | | | | | | | | | | | | | | | | | | | | | |
| **Infant** |  | |  | | 23F (4413) | |  | |  | | 23F (4413) | | 23F (4413) | | 23F (4413) | | 23F (4413) | |  | | 23F (4413) |  |
| **Mother** |  | |  | |  | |  | | 23F (4413) | |  | |  | |  | |  | |  | | 23F (4413) |  |
